# Vitamin D3 Regulates Energy Homeostasis under Short-Term Fasting Condition in Zebrafish (Danio Rerio)

**DOI:** 10.3390/nu16091271

**Published:** 2024-04-25

**Authors:** Qingyang Du, Rui Shao, Wentao Wang, Hui Zhang, Xinmeng Liao, Zhihao Wang, Zhan Yin, Qinghui Ai, Kangsen Mai, Xiao Tang, Min Wan

**Affiliations:** 1 Key Laboratory of Aquaculture Nutrition and Feed, Ministry of Agriculture & Key Laboratory of Mariculture, Ministry of Education, College of Fisheries, Ocean University of China, Qingdao 266003, China; 2 State Key Laboratory of Freshwater Ecology and Biotechnology, Institute of Hydrobiology, Chinese Academy of Sciences, Wuhan 430072, China; 3 Division of Physiological Chemistry II, Department of Medical Biochemistry and Biophysics, Karolinska Institutet, 17177 Stockholm, Sweden

**Keywords:** vitamin D, glucose, GLP-1, gut microbiota, hypoglycemia

## Abstract

Vitamin D3 (VD3) is a steroid hormone that plays pivotal roles in pathophysiology, and 1,25(OH)2D3 is the most active form of VD3. In the current study, the crucial role of VD3 in maintaining energy homeostasis under short-term fasting conditions was investigated. Our results confirmed that glucose-depriving pathways were inhibited while glucose-producing pathways were strengthened in zebrafish after fasting for 24 or 48 h. Moreover, VD3 anabolism in zebrafish was significantly suppressed in a time-dependent manner under short-fasting conditions. After fasting for 24 or 48 h, zebrafish fed with VD3 displayed a higher gluconeogenesis level and lower glycolysis level in the liver, and the serum glucose was maintained at higher levels, compared to those fed without VD3. Additionally, VD3 augmented the expression of fatty acids (FAs) transporter cd36 and lipogenesis in the liver, while enhancing lipolysis in the dorsal muscle. Similar results were obtained in *cyp2r1*^−/−^ zebrafish, in which VD3 metabolism is obstructed. Importantly, it was observed that VD3 induced the production of gut GLP-1, which is considered to possess a potent gluconeogenic function in zebrafish. Meanwhile, the gene expression of proprotein convertase subtilisin/kexin type 1 (pcsk1), a GLP-1 processing enzyme, was also induced in the intestine of short-term fasted zebrafish. Notably, gut microbiota and its metabolite acetate were involved in VD3-regulated pcsk1 expression and GLP-1 production under short-term fasting conditions. In summary, our study demonstrated that VD3 regulated GLP-1 production in zebrafish by influencing gut microbiota and its metabolite, contributing to energy homeostasis and ameliorating hypoglycemia under short-term fasting conditions.

## 1. Introduction

All animals obtain energy from food for growth, metabolism, reproduction and physical activities. When food is unavailable due to reasons such as population competition, seasonal alternation or reproductive behavior, animals have to use their internal energy reserves to survive [1]. Fasting is a metabolic state in which the body undergoes a period without food intake. Generally, fasting is divided into three phases according to the main energy substrates, and the energy metabolism strategies through the period of each phase vary among species. In mammals, serum glucose and glycogen storage are first utilized during phase 1. As fasting time increases, lipid reserves in adipose tissues commence lipolysis and release free fatty acids (FFAs), which are absorbed by the liver and used for energy supply during phase 2. The absorption process is associated with a variety of proteins named FA translocase and transport proteins, such as CD36 and solute carrier family 27A [2]. When lipid reserves are exhausted after prolonged fasting, phase 3 occurs and the protein reserves will be utilized for energy production [3]. In fish, the utilization of main nutrients under a fasting condition resembles that of mammals [4,5], although some fish species are able to endure longer fasting periods [6,7].

During short-term fasting, several hormones, including insulin, glucagon and GLP-1, play an important role in maintaining the homeostasis of glucose and lipid metabolism [8]. It is known that the serum concentration of insulin decreases while that of glucagon increases when mammals undergo fasting, leading to a high G/I (glucagon/insulin) molar ratio. In contrast to mammals, the G/I molar ratio in fasted fish remains at a low level even after long-term fasting [7], although the general functions of insulin and glucagon in fish resemble other vertebrates [9]. Additionally, GLP-1 is co-encoded with glucagon in the pro-glucagon gene [10]. Furthermore, pro-glucagon is processed by PC1/3 (encoded by *pcsk1*) to produce GLP-1 in intestinal L-cells, and by PC2 (encoded by *pcsk2*) to produce glucagon in pancreatic α-cells [10,11]. In mammals, GLP-1 increases insulin and decreases glucagon secretion in a glucose-dependent manner, thus improving glucose homeostasis and ameliorating metabolic diseases, such as diabetes and obesity [12,13,14,15]. Notably, it was proven that GLP-1 in fish exerted different functions in regulating glucose homeostasis from that in mammals [16,17].

VD_3_ is a steroid hormone that plays important roles in mineral homeostasis, metabolism, immunity, inflammation, and gut microbiota [18]. To be converted to its active form 1,25(OH)_2_D_3_, VD_3_ must undergo two-step hydroxylation by the enzymes 25-hydroxylase and 1-alpha-hydroxylase that are encoded by *cyp2r1* and *cyp27b1*, respectively [19]. 1,25(OH)_2_D_3_ acts primarily with the help of the nuclear vitamin D receptor (VDR) and can be deactivated by 24-hydroxylase (encoded by *cyp24a1*) [20,21]. Interestingly, it was reported that VD_3_ metabolism was influenced in fasted animals. For example, the expression level of *cyp2r1* and the enzyme activity of 25-hydroxylase in mice was significantly reduced, while the expression of *cyp24a1* was up-regulated after fasting for 24 h, suggesting short-term fasting suppressed 1,25(OH)_2_D_3_ biosynthesis and accelerated 1,25(OH)_2_D_3_ deactivation [22]. Similar results were reported in humans after 8 days of fasting [23].

The previous results from our research group demonstrated that VD_3_ lowered postprandial blood glucose levels in zebrafish under a hyperglycemia condition [24,25]. Moreover, Peng et al. showed that VD_3_ promoted FA oxidation in zebrafish adipose tissue [26]. Interestingly, it was proposed that *cyp2r1*^−/−^ zebrafish, in which VD_3_ metabolism is obstructed, failed to utilize lipid reserves to provide energy after long-term fasting [26]. Nonetheless, little has been known about the function of VD_3_ under short-term fasting conditions. Therefore, in the present study, we attempted to investigate the effect of the function of VD_3_ on the regulation of glucose and lipid metabolism under short-term fasting conditions by using zebrafish as an animal model.

## 2. Materials and Methods

### 2.1. Zebrafish Maintenance

The zebrafish were maintained at 28.5 °C with a 14 h light/10 h dark rhythm in a circulating aquarium system located in Fisheries College, Ocean University of China. The fish were fed twice daily with newly hatched brine shrimp (Artemia franciscana). Embryos were obtained by natural spawning and kept in egg water at 28.5 °C after 24 h incubation with 1% methylene blue. The generation of *cyp2r1^−/−^* zebrafish has been described in a previous report [26]. According to standard methods, the developmental stages were defined by day post-fertilization (dpf) or month post-fertilization (mpf).

The husbandry and handling of the fish were performed based on the Management Rule of Laboratory Animals (Chinese order no. 676 of the State Council, revised 1 March 2017). All animal procedures in the present study were approved by the Experimental Animal Ethics Committee at Ocean University of China.

### 2.2. Experimental Diets and Feeding Trial

Two experimental diets with supplementation of 800 or 0 IU VD_3_/kg were designed. The composition of the two diets is listed in Table 1. Before the formal feeding trial, wild-type zebrafish at 2 mpf were kept in eight tanks (5 L, 16 fish/tank), and fed the diet containing 0 IU/kg VD_3_ diet three times daily for one week. Thereafter, the fish were separated into two groups fed the VD_3_-containing or non-VD_3_ diet with four replicates for each group. In addition, the female-to-male ratio was adjusted to approximately 1:2 in each tank. All fish were fed 3 times at 8:30, 14:30, and 20:30 for three weeks. The waste and remaining feed were cleaned up and half of the water was renewed daily.

### 2.3. Antibiotic Treatment

The method of antibiotic treatment has been described previously [27]. In brief, wild-type zebrafish at 2 mpf were randomly allocated into 2 groups, and fed with the VD_3_ or non-VD_3_ diet for one month. In the meantime, four antibiotics (100 μg/mL ampicillin, 10 μg/mL kanamycin, 0.5 μg/mL amphotericin B, and 50 μg/mL gentamycin) were added to the water. The water containing antibiotics was renewed daily.

### 2.4. Sample Collection

Corresponding to the fasting period, the VD_3_ and non-VD_3_ groups were fasted for 0 h to 80 h after the last feeding before sampling. The fish were euthanized with 0.1% MS-222 (E10505, Sigma, Livonia, MI, USA). The blood sample was drawn from the caudal vessels of zebrafish according to a protocol previously described [28]. The blood samples from 3~4 fish were combined and centrifuged at 1000× *g* for 20 min. The supernatants were collected and immediately saved at −80 °C. In addition, the liver and gut from each fish were rapidly isolated and stored at −80 °C for further analysis.

### 2.5. RNA Extraction and Quantitative Real-Time PCR

The RNA of the liver and gut was extracted using the RNAeasyTM Animal RNA isolation kit (Beyotime, Shanghai, China) following the manufacturers’ instructions. The quantity and quality of total RNA were determined by using NanoDrop^®^ One spectrophotometer (Thermo Fisher Scientific, Waltham, MA, USA). Total RNA (1 μg) was used for reverse transcription with the HiScript III 1st Strand cDNA Synthesis Kit (R323-01, Vazyme, Nanjing, China). Quantitative RT–PCR was performed using the ChamQ Universal SYBR qPCR Master Mix kit on a quantitative thermal cycler CFX96TM Real-Time System (Bio-Rad, Hercules, CA, USA). The mRNA levels were calculated by the 2^−ΔΔCt^ method as the fold expression relative to the housekeeping gene, actb2. All primer sequences are listed in Table 2.

### 2.6. Measurement of Glucose, GLP-1 and 1,25(OH)_2_D_3_ Contents

The contents of glucose, GLP-1 and 1,25(OH)_2_D_3_ in zebrafish serum, gut or dorsal muscle were assessed by using the ELISA assay kits from Applygen Technologies (Cat. No. E1011, Beijing, China) and Hengyuan Biological Technology (Cat. No. HB299-QT and HB274-QT, Shanghai, China), according to the manufacturer’s instructions.

### 2.7. GF Zebrafish Generation and SCFA Treatment

GF zebrafish were obtained following an established method with some adjustments [29]. In brief, natural spawning zebrafish embryos were collected and incubated in GZM with antibiotics (100 μg/mL ampicillin, 10 μg/mL kanamycin, 0.5 μg/mL amphotericin B, 50 μg/mL gentamycin) at 28.5 °C for 6–8 h. Then, the embryos were treated with 0.1% PVP-I for 90 s, 0.003% bleach solution for 20 min, and rinsed twice with GZM. Tryptic soy agar plate, nutrient broth, brain–heart infusion broth, and Sab-Dex broth were used for sterility verification.

At 3 dpf, gnotobiotic zebrafish larvae were incubated with 30 mM sodium acetate, sodium propionate, or sodium butyrate for 2 days under sterile environments. Afterward, zebrafish were collected for further analysis.

### 2.8. Calculations and Statistical Analysis

The results are presented as means ± SEM. Raw data were analyzed by one-way or two-way ANOVA followed by Tukey’s multiple comparisons test after normality and homogeneity of variance were verified. Statistical analysis was performed using GraphPad Prism 9.5 (GraphPad Software, Inc., La Jolla, CA, USA), and *p* < 0.05 was considered as statistical significance.

## 3. Results

### 3.1. Short-Term Fasting Influences Glucose and Lipid Metabolism in Zebrafish

**Figure 1:**
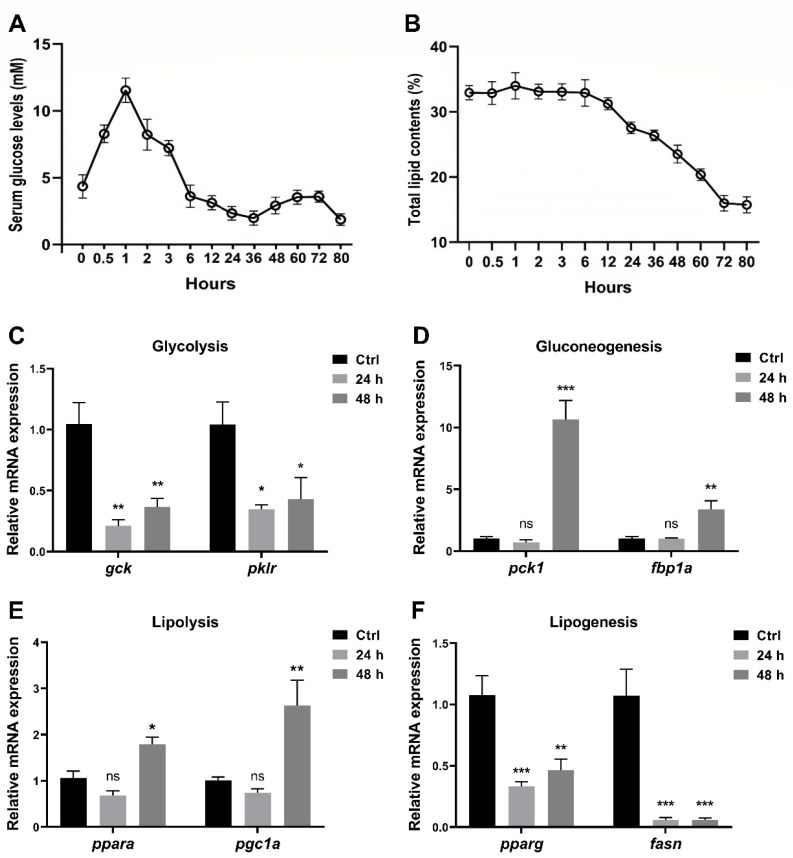
Short-term fasting influences glucose and lipid metabolism in zebrafish. WT zebrafish at 3 mpf were fasted for 0 h to 80 h after the last feeding. (**A**,**B**) The serum glucose levels (**A**) and total lipid contents in the whole fish (**B**) were evaluated (*n* = 3 replicates, 3 fish/replicate). (**C**–**F**) Relative expression levels of glycolysis-related genes (*gck* and *pklr*) (**C**), gluconeogenesis-related genes (*pck1* and *fbp1a*) (**D**), lipolysis-related genes (*ppara* and *pgc1a*) (**E**), and lipogenesis-related genes (*pparg* and *fasn*) (**F**) in the liver were determined (*n* ≥ 4/group). * *p* < 0.05, ** *p* < 0.01, *** *p* < 0.001, ns: no statistical significance.

After the last feeding, zebrafish were fasted for a desired time from 0 h to 80 h before being euthanized, and the serum glucose level and total lipid content were measured. As the results showed, the postprandial serum glucose levels increased, peaked around one hour, and gradually decreased to the initial level after six hours (Figure 1A). In contrast, the lipid content showed no significant changes during the first six hours (Figure 1B). Furthermore, the postprandial serum glucose levels declined mildly between 6 h and 36 h, when the lipid content started to decrease (Figure 1A,B). Interestingly, the postprandial serum glucose rose again between 36 h and 72 h, while it declined again after 72 h when the lipid content stopped decreasing (Figure 1A,B). Compared to the gene expression in the control group (one hour after feeding), the expression levels of glycolysis-related genes (*gck*, *pklr*) in zebrafish liver significantly decreased at 24 h and 48 h after feeding (Figure 1C), while gluconeogenesis-related genes (*pck1*, *fbp1a*) increased at 48 h after feeding (Figure 1D). In addition, the expression levels of lipolysis-related genes (*ppara*, *pgc1a*) were enhanced at 48 h (Figure 1E), and those of lipogenesis-related genes (*pparg*, *fasn*) were suppressed at 24 h and 48 h (Figure 1F).

### 3.2. 1,25(OH)_2_D_3_ Generation in Zebrafish Was Impaired under Short-Term Fasting Condition

**Figure 2:**
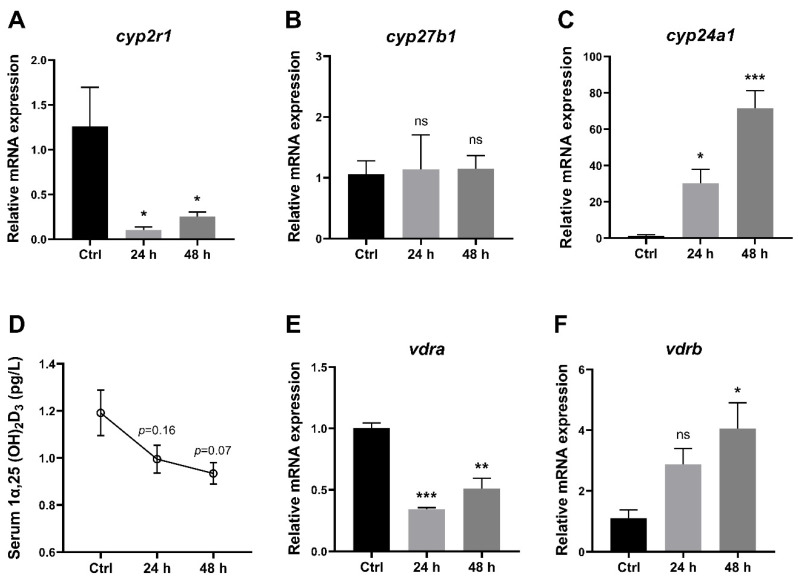
1,25(OH)_2_D_3_ generation in zebrafish was impaired under short-term fasting condition. (**A**–**C**). The gene expression of *cyp2r1* (**A**), *cyp27b1* (**B**) and *cyp24a1* (**C**) in zebrafish liver was assessed after fasting for 24 h or 48 h, compared to the control group (1 h postprandial) (*n* = 4/group). (**D**) The serum 1,25(OH)_2_D_3_ concentrations were determined (*n* = 3 replicates, 4~5 fish/replicate). (**E**,**F**) Gene expression levels of *vdra* (**E**) and *vdrb* (**F**) in the liver (*n* = 4/group). * *p* < 0.05, ** *p* < 0.01, *** *p* < 0.001, ns: no statistical significance.

To understand the effects of short-term fasting on VD_3_ metabolism in zebrafish, several key genes related to VD_3_ metabolism in the liver as well as 1,25(OH)_2_D_3_ concentrations in the serum were analyzed. The data showed that compared to the control group (one hour after feeding), the gene expression of *cyp2r1* was suppressed after 24 h and 48 h (Figure 2A), whereas *cyp27b1* showed no significant changes (Figure 2B). Meanwhile, the gene expression of *cyp24a1* was significantly elevated at 24 h and 48 h after feeding (Figure 2C). Moreover, 1,25(OH)_2_D_3_ concentrations in serum displayed a decreasing trend during the 48 h fasting period (Figure 2D). Interestingly, short-term fasting inhibited the expression of *vdra*, whereas it enhanced the expression of *vdrb* in the liver (Figure 2E,F). These results suggested that short-term fasting has a remarkable impact on the VD_3_ metabolism and signaling in zebrafish.

### 3.3. VD_3_ Regulates Glucose Metabolism in Zebrafish under Short-Term Fasting Condition

**Figure 3:**
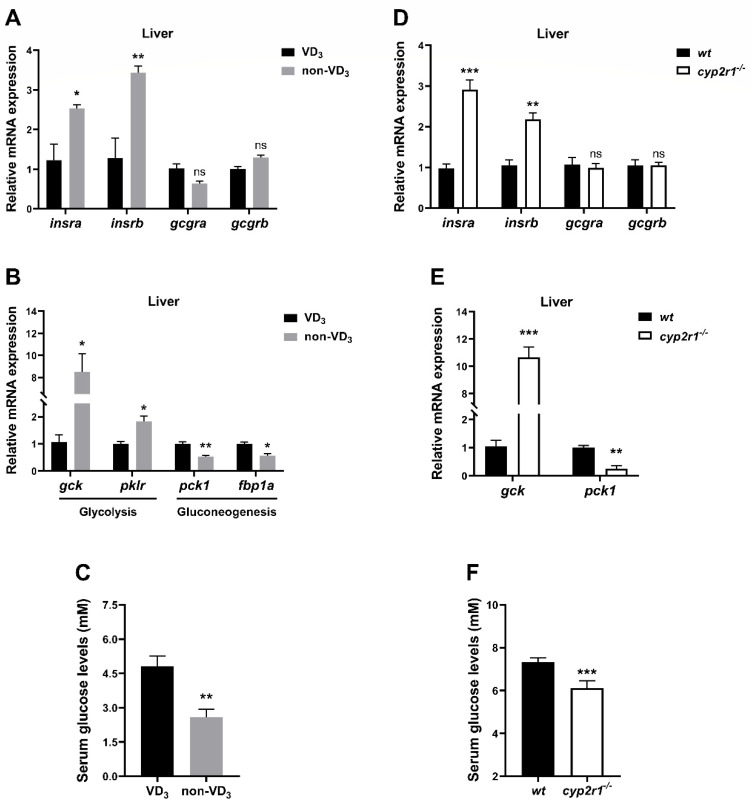
VD regulates glucose metabolism in zebrafish under short-term fasting conditions. (**A**–**C**) After the feeding trial, zebrafish fed with VD_3_ or non-VD_3_ diet were fasted for 24 h before sampling. The gene expression of *insra*, *insrb*, *gcgra*, *gcgrb* (**A**) and *gck*, *pklr*, *pck1*, *fbp1a* (**B**) in the liver was measured (*n* = 4/group). Serum glucose levels were determined (**C**) (*n* = 3~4 replicates, 4~5 fish/replicate). (**D**–**F**) WT and *cyp2r1*^−/−^ zebrafish at 3 mpf were fasted for 24 h before sampling. The gene expression of *insra*, *insrb*, *gcgra*, *gcgrb* (**D**) and *gck*, *pck1* (**E**) in the liver was analyzed (*n* = 4/genotype). Serum glucose levels were determined (**F**) (*n* = 4 replicates, 3 fish/replicate). * *p* < 0.05, ** *p* < 0.01, *** *p* < 0.001, ns: no statistical significance.

To investigate the link between 1,25(OH)_2_D_3_ and metabolic changes under fasting conditions, WT zebrafish were fed with a VD_3_ diet or non-VD_3_ diet for one month and fasted for 24 h before sacrifice. The gene expression levels of insulin receptors (*insra*, *insrb*) in the liver were significantly enhanced in the non-VD_3_ group. Notably, no significant changes were detected in glucagon receptors (*gcgra*, *gcgrb*) (Figure 3A). Lack of dietary VD_3_ led to an increase in the gene expression of glycolysis-related genes (*gck*, *pklr*), and a decrease in gluconeogenesis-related genes (*pck1*, *fbp1a*) (Figure 3B). Moreover, the serum glucose level of the non-VD_3_ group zebrafish was significantly lower than that of the VD_3_ group under short-term fasting conditions (Figure 3C). Additionally, WT and *cyp2r1*^−/−^ zebrafish at 3 mpf were fasted for 24 h. As anticipated, the gene expression of *insra*, *insrb*, *gcgra*, and *gcgrb* (Figure 3D) as well as the glycolysis-related gene (*gck*) and gluconeogenesis-related gene (*pck1*) (Figure 3E) in *cyp2r1*^−/−^ zebrafish liver followed a similar pattern as those in the non-VD_3_ group. Moreover, the serum glucose level was significantly lower in *cyp2r1*^−/−^ zebrafish after 24 h of fasting (Figure 3F). In addition, triglyceride levels in the serum and dorsal muscle were measured. Although there was an increasing trend of the triglyceride level in the dorsal muscle, the serum TG contents remained unchanged (Supplemental Figure S1).

### 3.4. VD_3_ Regulates Lipid Metabolism in Zebrafish under Short-Term Fasting Condition

**Figure 4:**
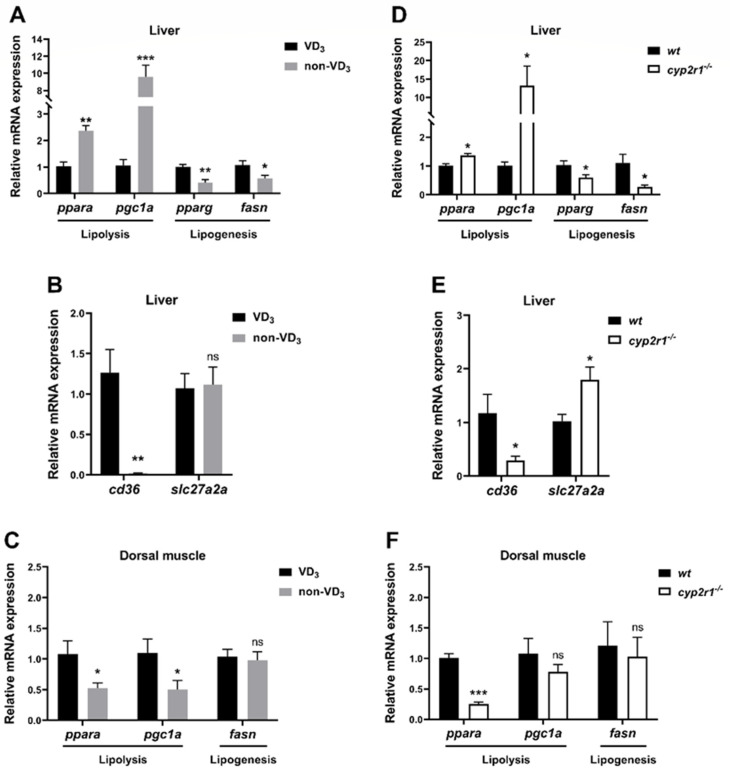
VD regulates lipid metabolism in zebrafish under short-term fasting conditions. (**A**–**C**) After the last feeding, zebrafish fed with VD_3_ or non-VD_3_ diet were fasted for 24 h before sampling. The gene expression of *ppara*, *pgc1a*, *pparg*, *fasn* (**A**) (*n* = 4/group), and FFA absorption-related genes *cd36*, *slc27a2a* (**B**) (*n* = 5/group) in the liver were determined. The gene expressions of *ppara*, *pgc1a*, *fasn* in the dorsal muscle were analyzed (**C**) (*n* = 6/group). (**D**–**F**) WT and *cyp2r1*^−/−^ zebrafish at 3 mpf were fasted for 24 h before sampling. The gene expressions of *ppara*, *pgc1a*, *pparg*, *fasn* (**D**) (*n* = 4/genotype), and *cd36*, *slc27a2a* (**E**) (*n* = 4/genotype) in the liver were determined. The gene expressions of *ppara*, *pgc1a*, *fasn* in the dorsal muscle were determined (**F**) (*n* = 4/genotype). * *p* < 0.05, ** *p* < 0.01, *** *p* < 0.001, ns: no statistical significance.

Compared to the VD_3_ group, the expression levels of lipolysis-related genes (*ppara* and *pgc1a*) were significantly elevated in the non-VD_3_ group, while the lipogenesis-related genes (*pparg* and *fasn*) decreased (Figure 4A). The expression of the FA absorption-related gene *cd36* in the liver was significantly suppressed, while the gene expression of *slc27a2a* showed no significant changes (Figure 4B). In contrast, the gene expression of *ppara* and *pgc1a* in the dorsal muscle was inhibited in the non-VD_3_ group, while *fasn* showed no significant changes (Figure 4C). In addition, WT and *cyp2r1*^−/−^ zebrafish at 3 mpf were fasted for 24 h. Compared to WT zebrafish, the expression levels of lipolysis-related genes (*ppara* and *pgc1a*) were enhanced while those of lipogenesis-related genes (*pparg* and *fasn*) were suppressed after 24 h of fasting (Figure 4D). Meanwhile, the gene expression of *cd36* was significantly reduced, though *slc27a2a* was increased in *cyp2r1*^−/−^ zebrafish after 24 h fasting (Figure 4E). Similarly, the expression of the lipolysis-related gene *ppara* was decreased in the dorsal muscle, while that of *pgc1a* and *fasn* showed no significant changes (Figure 4F). These results further confirmed that VD_3_ plays a crucial role in regulating lipid metabolism in zebrafish under short-term fasting.

### 3.5. VD_3_ Promotes the Synthesis and Processing of GLP-1 in the Gut under Short-Term Fasting Condition

**Figure 5:**
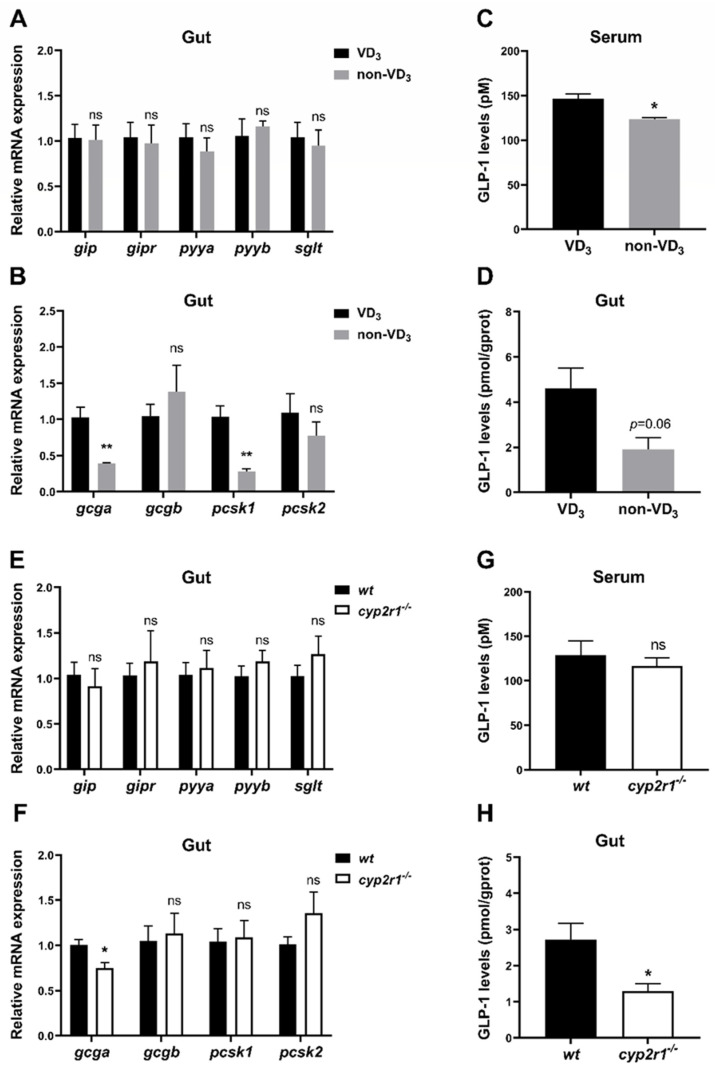
VD_3_ promotes the synthesis and processing of GLP-1 in the gut under short-term fasting conditions. (**A**–**D**) Zebrafish were fed with or without VD_3_ for a month and fasted for 24 h. The gene expression of *gip*, *gipr*, *pyya*, *pyyb*, *sglt* (**A**) and *gcga*, *gcgb*, *pcsk1*, *pcsk2* (**B**) in the gut was analyzed (*n* = 4/group). GLP-1 levels in the serum (**C**) and the intestine (**D**) were measured (*n* = 3 replicates, 4~5 fish/replicate). (**E**–**H**) WT and *cyp2r1*^−/−^ zebrafish at 3 mpf fasted for 24 h. The gene expression of *gip*, *gipr*, *pyya*, *pyyb*, *sglt* (**E**), and *gcga*, *gcgb*, *pcsk1*, *pcsk2* (**F**) in the intestine was analyzed (*n* = 5/genotype). GLP-1 levels in the serum (**G**) (*n* = 4 replicates, 3 fish/replicate) and the intestine (**H**) (*n* = 3/ genotype) were measured. * *p* < 0.05, ** *p* < 0.01, ns: no statistical significance.

Considering the significant impact of gastrointestinal tract-derived hormone peptides on lipid and carbohydrate metabolism [30], we further verified whether these hormones participated in regulating energy metabolism by VD_3_ during short-term fasting. The results showed no significant differences in the expression levels of *gip*, *gipr*, *pyya*, *pyyb*, and *sglt* between VD_3_ and non-VD_3_ groups (Figure 5A). However, the gene expression of *gcga* was significantly suppressed when zebrafish were fed with a non-VD_3_ diet, and the gene expression of *pcsk1* in zebrafish intestine was significantly reduced in the non-VD_3_ group compared to the VD_3_ group, while *gcgb* and *pcsk2* displayed no significant changes (Figure 5B). As expected, the level of GLP-1, the product of the *gcg* gene, was lower in the non-VD_3_ group compared to the VD_3_ group, both in fish serum (Figure 5C) and the gut (Figure 5D). Moreover, similar results were obtained in the *cyp2r1*^−/−^ zebrafish compared to WT zebrafish, except that no significant changes were detected in the serum GLP-1 level (Figure 5E) or the gene expression of *pcsk1* in the gut (Figure 5H).

### 3.6. Interaction between VD_3_ and Gut Microbiota under Short-Term Fasting Condition

**Figure 6:**
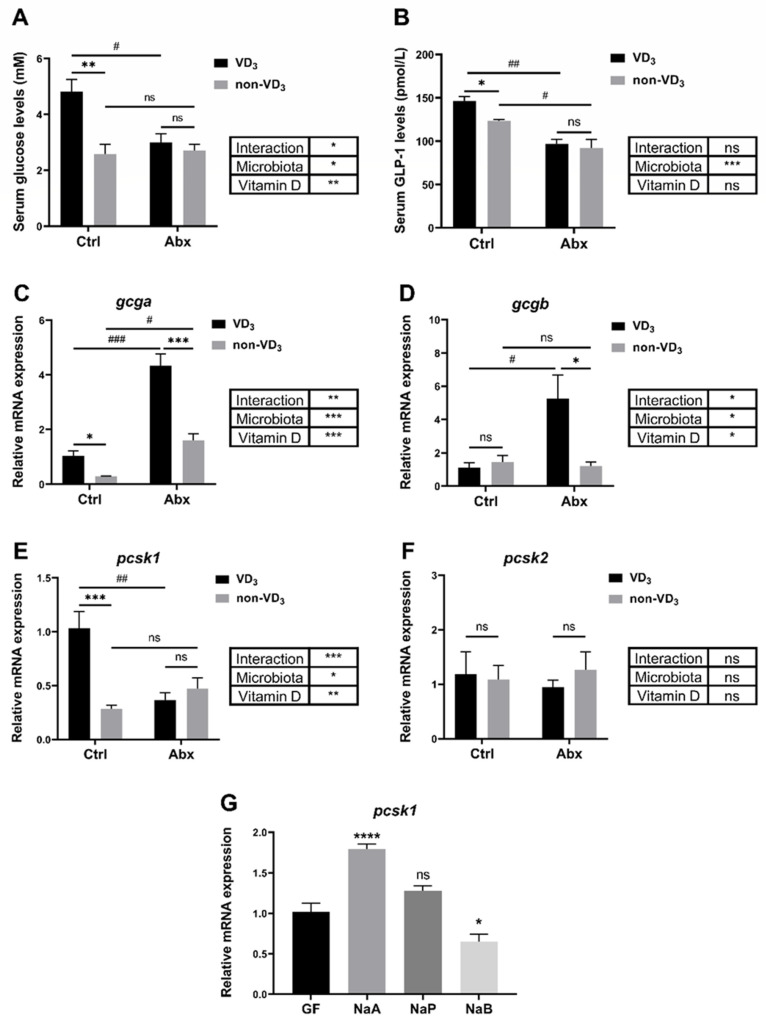
Interaction between VD3 and gut microbiota under short-term fasting conditions. (**A**–**F**) Zebrafish were treated with an antibiotic cocktail (Abx) while fed a VD3 or non-VD3 diet for one month. The serum glucose levels (**A**) and serum GLP-1 levels (**B**) were assayed (*n* = 3 replicates, 4~5 fish/replicate). The expression levels of gcga (**C**), gcgb (**D**), pcsk1 (**E**) and pcsk2 (**F**) in the intestine were analyzed (*n* = 4/group). (**G**) GF zebrafish at 3 dpf were incubated with NaA, NaP, or NaB (30 mM) for two days. The gene expression of pcsk1 was determined (*n* = 5 replicates, 10 larvae/replicate). * *p* < 0.05, ** *p* < 0.01, *** *p* < 0.001, **** *p* < 0.0001, # *p* < 0.05, ## *p* < 0.01, ### *p* < 0.001, ns: no statistical significance.

Our recent study provided evidence that VD_3_ could exert its physiological functions by influencing gut microbiota [31]. Interestingly, the serum glucose level exhibited no significant increment in the zebrafish fed with a VD_3_ diet after fasting for 24 h when the gut microbiota was removed by an antibiotic cocktail treatment (Figure 6A). The serum GLP-1 was decreased by the depletion of microbiota, and the effects of VD_3_ on GLP-1 diminished without the microbiota under short-term fasting conditions (Figure 6B). Notably, VD_3_ deficiency still caused a significant reduction in the expression of *gcga* when the microbiota was depleted (Figure 6C). In contrast, VD_3_ deficiency strongly restrained the gene expression of *gcgb* in the gut of zebrafish under short-term fasting, although the regulation of *gcgb* by VD_3_ could not be detected without the depletion of gut microbiota (Figure 6D). Interestingly, the inhibition of *pcsk1* gene expression in the non-VD_3_ group was abolished after the depletion of microbiota (Figure 6E). The expression level of *pcsk2* showed no evident response to both VD_3_ and microbiota (Figure 6F). Importantly, germ free (GF) zebrafish were incubated with short-chain fatty acids, including acetate, propionate and butyrate, which are crucial metabolites of gut microbiota [32]. The result showed that the gene expression of *pcsk1* in zebrafish was significantly enhanced by acetate treatment (Figure 6G). Altogether, our data confirmed that the microbiota is required in the regulation of GLP-1 production by VD_3_ under short-term fasting conditions.

## 4. Discussion

In the present study, we have demonstrated that VD contributes to global changes in glucose and lipid metabolism and ameliorates energy homeostasis under short-term fasting conditions. On one hand, VD promotes GLP-1 production that is helped by gut microbiota, resulting in higher gluconeogenesis and lower glycolysis levels in the liver, which maintains higher serum glucose levels in short-term fasted zebrafish. On the other hand, VD seems to enhance lipid mobilization for energy supply under short-term fasting conditions.

During fasting, the energy for animal survival comes from the reserved nutrients including glycogens, lipids and proteins. Though influenced by many factors, glucose is regarded as the primary energy substance during early fasting [8]. It has been known that major glucose-depriving pathways are glycolysis, glycogenesis and lipogenesis, whereas glucose-producing pathways are glycogenolysis and gluconeogenesis [33]. Our data confirmed that glucose-depriving pathways were suppressed while glucose-producing pathways were promoted after fasting for 48 h, which is associated with the second wave of the serum glucose level. Interestingly, our results demonstrated that VD contributed to maintaining the blood glucose concentrations at appropriate levels by further down-regulating glucose-depriving pathways and enhancing glucose-producing pathways under short-term fasting conditions. Notably, 1,25(OH)_2_D_3_ generation in zebrafish was impaired under short-term fasting conditions, which could have an adverse influence on maintaining glucose homeostasis.

It is well-known that glucose and lipid metabolism are correlated in every facet [34]. The association between FAs and gluconeogenesis was discovered in the 1990s [35]. Moreover, the mechanism through which insulin inhibits gluconeogenesis by suppressing adipose tissue lipolysis was confirmed recently [36]. As the pivot organ of lipid metabolism, the liver absorbs FFAs from food digestion, synthesizes triglycerides, and transports them to other target organs for lipid storage or energy supply [34]. During fasting, foodborne FFAs run out and the liver absorbs FFAs produced by lipid-storage organs, such as abdominal fat and intramuscular fat [37]. Most of the FFAs are transported into the hepatocyte with the help of FA translocase and FA transport proteins, such as CD36 and solute carrier family 27A. Our data showed that VD deficiency significantly enhanced lipolysis and suppressed lipogenesis in the liver while restraining the lipolysis pathway in the dorsal muscle during short-term fasting. Notably, the expression of *cd36* was dramatically suppressed in the liver of VD-deficient zebrafish. It is known that CD36 is a critical fatty acid sensor and regulator of lipid metabolism [38], and it was reported that CD36 expression in murine liver increased during fasting possibly to enhance the hepatic uptake of FA mobilized from other tissues [39]. Hence, the suppressed CD36 expression in the liver of VD-deficient zebrafish inferred that the lipid transportation from other tissues, such as dorsal muscles, to the liver might be dampened. Interestingly, it was reported that *cyp2r1*^−/−^ zebrafish failed to mobilize the fat storage in abdominal adipose tissue over 10 days or 35 days of fasting [26]. Meanwhile, serum FFA contents were higher in *cyp2r1*^−/−^ zebrafish, both under postprandial and fasting conditions [26], indicating the obstruction in FFA absorption and utilization. Hence, we conjectured that VD promoted lipid mobilization and energy supplements during short-term fasting, leading to a more stable level of blood glucose.

In the present study, we demonstrated that VD_3_ induced GLP-1 production in zebrafish under short-term fasting conditions. GLP-1 is a hormone peptide secreted from intestinal L-cells and processed by PC1/3 (encoded by *pcsk1*) [10]. As previous reports showed, GLP-1 might interact with the central neuronal circuits involved in food intake control through the gut–brain axis [17,40]. Notably, GLP-1 in fish displayed opposite functions in regulating glucose metabolism, compared to those in mammals, which may be related to the genome duplication event and the depletion of the GLP-1 receptor in fish [41]. Importantly, we further identified that the gut microbiota was involved in VD-regulated *pcsk1* expression and GLP-1 production, although VD-regulated gene expression of *gcga* was independent of gut microbiota. Hence, it was possible that the processing of proglucagon to GLP-1 was influenced by the gut microbiota. Interestingly, previous studies have reported that VD_3_ influenced the composition of gut microbiota in humans and mice [42,43]; however, the holistic mechanism is still a mystery. Considering that the bacteria do not have VDR, it was conjectured that VD might influence the gut microbiota in indirect manners [44,45]. Nonetheless, our recent study uncovered that VD_3_ promoted the in vitro growth of certain probiotics directly [31].

On the other hand, short-chain fatty acids (SCFAs), including butyrate, propionate and acetate, are the main metabolites produced by gut microbiota [46]. Sanna et al. demonstrated that the level of circulating SCFAs was related to insulin sensitivity and GLP-1 concentration in humans [32]. Moreover, Kumar et al. reported that SCFAs significantly induced the gene expression of *pcsk1* in the STC-1 cells [47]. Recently, we discovered that VD significantly increased the relative abundance of *Cetobacterium* spp. in the gut microbiota of zebrafish, as well as the serum concentration of acetate, a major product of *Cetobacterium* spp. [31]. In the present study, we confirmed that acetate rather than propionate or butyrate enhanced the gene expression of *pcsk1*, suggesting that the up-regulation of GLP-1 production may be attributed to the VD-induced changes in the metabolite from gut microbiota.

It is noteworthy that the potential hypoglycemic role of VD_3_ in diabetes has attracted increasing attention in recent years [48,49]. Our research group has demonstrated that VD_3_ lowers postprandial blood glucose levels in zebrafish under hyperglycemia conditions [24,25]. Although diabetes patients suffer from postprandial hyperglycemia, they may encounter hypoglycemia caused by strict food control and the use of diabetes drugs, which has long been recognized as a major barrier to achieving normoglycemia for diabetic patients with intensive therapy [50,51]. Considering our results that proved VD alleviated hypoglycemia caused by short-term fasting in zebrafish, it would be very intriguing to validate the potential application of VD_3_ in the therapy of hypoglycemia in humans.

This study has demonstrated an unexpected role of VD_3_ in glucose metabolism under short-term fasting conditions, using zebrafish as a model. Considering zebrafish are a good model for physiological research, the results could shed light on both teleost and human research. Given that VD_3_ lowers postprandial blood glucose levels in zebrafish under hyperglycemia conditions [24,25], the current study provided clear evidence for the first time that this multifunctional hormone may exert varied effects on glucose metabolism in a different metabolic state, i.e., short-term fasting condition. However, the mechanistic link between VD_3_ supplement and lipid mobilization remains obscure in the present study. Further studies are warrantied to clarify this issue.

## 5. Conclusions

The present study uncovered the crucial role of VD_3_ in maintaining energy homeostasis in zebrafish under short-term fasting conditions. Importantly, VD_3_ promotes GLP-1 production in a gut microbiota-dependent manner, resulting in the alleviation of fasting-caused hypoglycemia. Our study emphasized the importance of sufficient VD_3_ in maintaining energy homeostasis, and highlighted the potential application of VD_3_ in the therapy of hypoglycemia and regulating glucose homeostasis.

## Figures and Tables

**Table 1:** Dietary formulation of experimental diets (g/kg).

Ingredient (g/kg)	Non-VD_3_ Diet	VD_3_ Diet
Casein	320	320
Gelatin	80	80
Corn starch	300	300
Soybean oil	70	70
Choline chloride	5	5
Monocalcium phosphate	20	20
Carboxymethyl cellulose	21	21
Sodium alginate	6	6
Vitamin premix ^a^ (VD_3_-free)	10	10
Mineral premix ^b^	30	30
Cellulose	138	138
Vitamin D_3_ (IU/kg)	0	800
Total	1000	1000
Proximate composition (dry matter basis %)		
Moisture	8.64	8.36
Crude protein	35.7	35.7
Crude lipid	5.11	5.12
Ash	3.65	3.63

^a^ Vitamin premix (mg/g diet): thiamin hydrochloride, 5; riboflavin, 10; calcium pantothenate, 10 mg; D-biotin, 0.6; pyridoxine hydrochloride, 4; folic acid, 1.5; inositol, 200; L-ascorbyl-2-monophosphate-Mg, 60; nicotinic acid, 6.05; α-tocopheryl acetate, 50; menadione, 4; retinol acetate, 2000 IU. All ingredients were diluted with micro-cellulose to 1 g. ^b^ Mineral mixture supplied the following (mg/g diet): Ca (H_2_PO_4_)_2_·H_2_O,135.8; Ca (CH_3_CHOHCOO)_2_·5H_2_O, 327; FeSO_4_·6H_2_O, 2.125; MgSO_4_·7H_2_O, 137; NaH_2_PO_4_, 87.2; NaCl, 43.5; AlCl_3_·6H_2_O, 0.15; KIO_3_, 0.125; KCl, 75; CuCl_2_·2H_2_O, 0.1; MnSO_4_·H_2_O, 0.80; CoCl_2_·6H_2_O, 1 and ZnSO_4_·7H_2_O, 3. All ingredients were diluted with micro-cellulose to 1 g.

**Table 2:** Primer sequences for qRT-PCR.

Gene	NCBI Accession No.	Forward (5′-3′)	Reverse (5′-3′)
*hk1*	NM_213252.1	ACTTTGGGTGCAATCCTGAC	AGACGACGCACTGTTTTGTG
*gck*	NM_001045385.2	TGAGGATGAAGAGCGAGGC	AGAGAAGGTGAATCCCAGCG
*pklr*	NM_201289.1	CAAAGGACACTTCCCTGTAGAG	GGACAACGAGGACGATAACG
*pck1*	NM_214751.1	GTGAACTGAACCGAGACCTG	AGCACTTGAGAGCAAACGAT
*fbp1a*	NM_199942.2	CATCTGTATGGGATTGCTGG	TTACCCCGTCTATCTGGCTC
*g6p1a.1*	NM_001003512.2	GCTGCACCATACGAGATGGA	TCACCAAACAGCACCCACTT
*ppara*	NM_0 01161333.1	TGCTGGACTACCAGAACTGTGACA	TGCTGGCTGAGAACACTTCTGAG
*pgc1a*	XM_017357138.1	AGTCTCCAAATGACCACAAGG	GGTTCTCTTGACTGGCTTTGT
*pparg*	NM_131467.1	TTTTCCGCAGGACGATT	GAGGGAAGTATTTGAGATAGGAC
*fasn*	XM_021472581.1	GGAGAATCTGACCCCACA	CTCCAAAACGACACCCAC
*cyp2r1*	NM_001386362.1	TGGAGAACTGATCATCGCGG	CCTCCACATACGGCATCCTC
*cyp27b1*	NM_001311791.1	AAGGCCGTCGTCAAGGAAAT	CTCGAGACGTGGCGTAATGA
*cyp24a1*	NM_001089458.1	CCTCCACATACGGCATCCTC	CCAAACGGCACATGAGCAAA
*vdra*	NM_130919.1	GGATTCCACTTCAACGCC	CTCAGCCGAGGTTTACGA
*vdrb*	NM_001159985.1	CAGTATGAAGCGGAAGGC	GGAGGTCTGAAGCGTGAA
*insra*	NM_001142672.1	GGTGGGTGACAGGGTTCTTT	GCACACAGTCCGGATAACCT
*insrb*	NM_001123229.1	TCATTTCACCCCTGCTGTGT	AGCAGCCGAAGTCTACATGG
*gcgra*	XM_021474732.1	ATGAGCAGAGAAGCACCGAT	CAGGATGAAGGAGGCAAACA
*gcgrb*	XM_009295263.3	CCACTACCAGAGCACACGAT	ACTCTTTGGGCACAGACTCA
*cd36*	NM_001002363.1	GCCTGTTGATGCTCTGGCTTCTC	CATTCCGACCACCCCCTGC
*slc27a2a*	NM_001025299.1	CGTGCTTCTCCACACTCGAT	TGCATCCCGGTAAGTGTAGC
*gip*	NM_001080059.1	TGCGCTGGTTTTGATTTGCC	TATCGGCGACTGAGCTTCTG
*gipr*	XM_005157739.4	TGAGTGGGAAGACGGTGAA	CGGCTCGCAGGATGAATG
*pyya*	NM_001164371.1	CGTCGCCACTGTCCTCA	TCCATACCGTTGCCTCGT
*pyyb*	NM_001327895.1	CCACCCAAACCTGAACCT	CAAGTCTTCAACACGAGGC
*sglt*	NM_200681.1	TAAAGCTGTCTGTGGAGCCGAAGT	ACAACATCAACCCTCGGAGACCAT
*gcga*	NM_001008595.3	TGCCAGTCTTCTTTTGCTCC	CAGGTATTTGCTGTAGTCGTTG
*gcgb*	NM_001242770.1	GGAGACCAGGAGAGCACAAG	TGCAGGTACGAGCTGACATC
*pcsk1*	NM_001137662.1	TTGGGCCGAACAGCAGTATGAGAAA	TGGATAAATGTCGGTGTGGTTCCAC
*pcsk2*	NM_001142266.1	CGCAAGAGAAACCCTGAAGC	TCTTGGAGGTCAGAACCGTC
*actb2*	NM_181601.5	GATGATGAAATTGCCGCACTG	ACCAACCATGACACCCTGATGT

## Data Availability

Original data in this study are available from the corresponding author according to reasonable request.

## Supplementary Materials

The following supporting information can be downloaded at: https://www.mdpi.com/article/10.3390/nu16091271/s1, Figure S1. Triglyceride levels in the serum and dorsal muscle of zebrafish. (A,B) After the feeding trial, zebrafish fed with VD3 or non-VD3 diet were fasted for 24 h before sampling. TG contents in the serum (A) were determined (*n* = 3~4 replicates, 4~5 fish/replicate), as well as dorsal muscle (B) (*n* = 4/group). (C) WT and cyp2r1^−/−^ zebrafish at 3 mpf were fasted for 24 hours before sampling. TG contents in the dorsal muscle were determined (*n* = 4/genotype). ns: no statistical significance

## Abbreviations

WT	wild type
FA	fatty acid
FFAs	free fatty acids
PC1/3	prohormone convertase 1/3
PC2	prohormone convertase 2
GLP-1	glucagon-like peptide 1
GF	germ-free
GZM	gnotobiotic zebrafish medium
SCFAs	short-chain fatty acids
NaA	sodium acetate
NaP	sodium propionate
NaB	sodium butyrate
hk1	hexokinase 1
gck	glucokinase
pklr	pyruvate kinase L/R
pck1	phosphoenolpyruvate carboxykinase 1
fbp1a	fructose-1,6-bisphosphatase 1a
g6p1a.1	glucose-6-phosphatase catalytic subunit 1a, tandem duplicate 1
ppara	peroxisome proliferator-activated receptor alpha
pgc1a	peroxisome proliferator-activated receptor gamma, coactivator 1 alpha
pparg	peroxisome proliferator-activated receptor gamma
fasn	fatty acid synthase
cyp2r1	cytochrome P450, family 2, subfamily R, polypeptide 1
cyp27b1	cytochrome P450, family 27, subfamily A, polypeptide 1
cyp24a1	cytochrome P450, family 24, subfamily A, polypeptide 1
vdra	vitamin D receptor a
vdrb	vitamin D receptor b
insra	insulin receptor a
insrb	insulin receptor b
gcgra	glucagon receptor a
gcgrb	glucagon receptor b
gip	gastric inhibitory polypeptide
gipr	gastric inhibitory polypeptide receptor
pyya	peptide YY a
pyyb	peptide YY b
sglt	sodium-glucose cotransporter 1
cd36	CD36 molecule
slc27a2a	solute carrier family 27 member 2a
gcga	glucagon a
gcgb	glucagon b
pcsk1	proprotein convertase subtilisin/kexin type 1
pcsk2	proprotein convertase subtilisin/kexin type 2
actb2	actin, beta 2
