# Supplementary material for: Vitamin D3 Regulates Energy Homeostasis under Short-Term Fasting Condition in Zebrafish (Danio Rerio)
Source: Nutrients. 2024 Apr 25;16(9):1271. doi: 10.3390/nu16091271 (PMC11085765; doi:10.3390/nu16091271)
Supplement: Supplementary file 1 [file nutrients-16-01271-s001.zip › nutrients-2922624-supplementary.pdf]

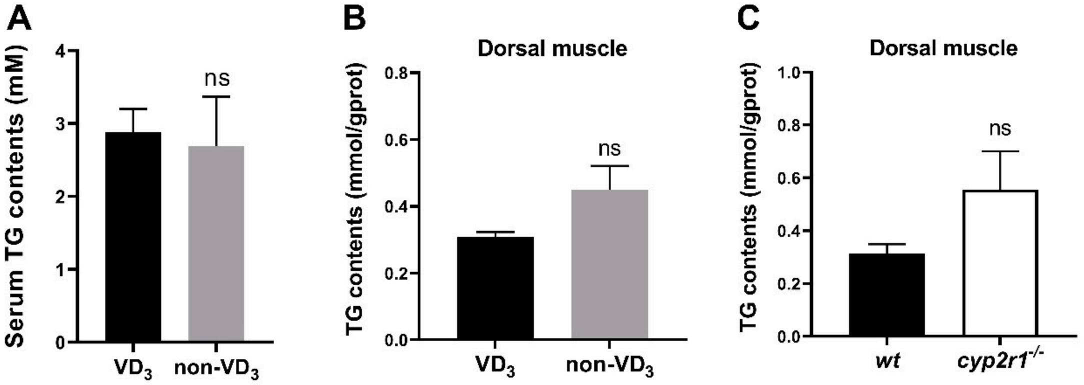

Supplemental Figure S1. Triglyceride levels in the serum and dorsal muscle of zebrafish. (A-B) After the feeding trial, zebrafish fed with VD_3 or non-VD_3 diet were fasted for 24 h before sampling. TG contents in the serum (A) were determined (n = 3~4 replicates, 4~5 fish/replicate), as well as dorsal muscle (B) (n = 4/group). **(C)** WT and *cyp2r1*$^{-/-}$ zebrafish at 3 mpf were fasted for 24 hours before sampling. TG contents in the dorsal muscle were determined (n = 4/genotype). ns: no statistical significance.
